# Self-sacrificed template synthesis of ribbon-like hexagonal boron nitride nano-architectures and their improvement on mechanical and thermal properties of PHA polymer

**DOI:** 10.1038/s41598-017-08524-7

**Published:** 2017-08-21

**Authors:** Yan Zhao, Zhenya Liu, Chaochao Cao, Chong Wang, Yi Fang, Yang Huang, Chao Yu, Jun Zhang, Lanlan Li, Long Hu, Chengchun Tang

**Affiliations:** 1 School of Materials Science and Engineering, Hebei University of Technology, Tianjin, 300130 P. R. China; 2 Hebei Key Laboratory of Boron Nitride Micro and Nano Materials, Hebei University of Technology, Tianjin, 300130 P. R. China

## Abstract

Two-dimensional (2-D) boron nitride (BN) nanomaterials have received intensive attention because of their attractive mechanical, thermal and chemical stability. Here we demonstrate, for the first time, the synthesis of ribbon-like hexagonal boron nitride nano-architectures (RLBN) through a simple self-sacrificed template method using the cheap boric acid and melamine as raw materials. After the freeze-drying and thermal decomposition process, uniform ultrathin RLBN with width of 200–500 nm and thick of a few nanometers can be obtained. The RLBN with high quality tremendously improves the mechanical and thermal properties of Polyhydroxyalkanoates (PHA) polymer. The decomposition temperature (Td) of PHA increases from 368 °C to 390 °C, while the thermal conductivity increases by 46.0% with RLBN doped. The ductility (strain at break), yield strength and tensile strength of PHA@RLBN composite are also enhanced by 52.3%, 49.4% and 6.01% respectively.

## Introduction

To date, high temperature pyrolysis^1–7^ have been widely used to fabricate desired various nanostructures with controlled morphology. Synthesis of various nanostructures of boron nitride (BN), such as activated BN^1^, three-dimensional BN foam^2^, porous BN^3^, ultrafine porous BN nanofibers^4^. BN nano spheres^5^, BN nanosheets^6^ and BN nanotubes^7^ were continuously reported. Since the discovery of graphene^8^, two-dimensional (2D) nanomaterials have drawn extensive attention as potential building blocks for future nano scaled devices^9^. Follow-up as well as some other two-dimensional materials (like BN) were separated out in succession. Wang *et al*.^10^ find a simple and novel “biomass-directed on site synthesis” method to fabricate large scale high quality boron nitride nanosheets. The use of BN nanosheets in epoxy resin exhibit higher properties than pure epoxy resin in terms of thermal conductivity.

The atomic structure of boron nitride and graphene is the same, but the boron nitride than graphene has better thermal stability and chemical inertness^10–12^. Therefore, two-dimensional boron nitride materials have more broad application prospects^13^ in packaging field.

Polyhydroxyalkanoates (PHA), as a novel plastic and packing material, has attracted the attention of academia and industry because of its unique physical characteristics such as complete bio-degradability, good biocompatibility, environmental protection, and plastic thermal processability^14, 15^. However, PHA possesses a low thermal property and mechanical property, similar as conventional plastics. Studies launched on improving the thermal and mechanical properties of PHA. Xian *et al*.^16^ find that the elongation to break, ultimate tensile strength and elastic module of PHA with 0.4% ZnO increase by 41.2%, 55% and 62.2%, respectively. But the thermal stability of ZnO reinforced PHA composites did not improve. BN materials, as frequently used fillers in polymers^17, 18^, were also considered as candidates of filler in PHA. Xue *et al*.^19^ find that with the addition of 2 wt% BN fibers, the decomposition temperatures of PHA composites increased by 12 °C, and the thermal conductivity increase by 27.3% at the same time.

In this study we demonstrate, for the first time, the synthesis of a novel ribbon-like hexagonal boron nitride nano-architectures (RLBN) through a simple self-sacrificed template method using the cheap boric acid and melamine as raw materials. The morphology of RLBN with high quality tremendously improves the thermal and machinery properties of PHA polymer.

## Results and Discussion

### Characterization of RLBN

The RLBN is fabricated through a two steps self-sacrificed template method. In the first step, ribbon-like M∙2B nanomaterials (Fig. S1) are generated via a freeze-drying process. The ribbon-like M∙2B nanomaterials work simultaneously as precursor and template. In the second step, the ribbon-like M∙2B nanomaterials *in*-*situ* pyrolysis into RLBN, with the morphology remaining unchanged via a self-sacrificed heat treatment process. The morphology of ribbon-like M∙2B nanomaterials remained unchanged during pyrolysis. Table 1 shows the B, N, O, and C contents of the as obtained sample analyzed by conventional elemental analyzers. The boron and nitrogen contents are 41.69 wt% and 42.30 wt% respectively, close to the stoichiometric ratio of boron nitride. Only 1.01 wt% of carbon left during the synthesis process. In addition, 15.00 wt% of oxygen content may attributes to hydroxyl, which can be confirmed by Fourier transform infrared (FTIR) result given later in this paper.

**Table 1:** B, N, C and O concentration of the as prepared RLBN sample.

Sample	B (wt%)	N (wt%)	C (wt%)	O (wt%)
RLBN	41.69	42.30	1.01	15.00

**Figure 1:**
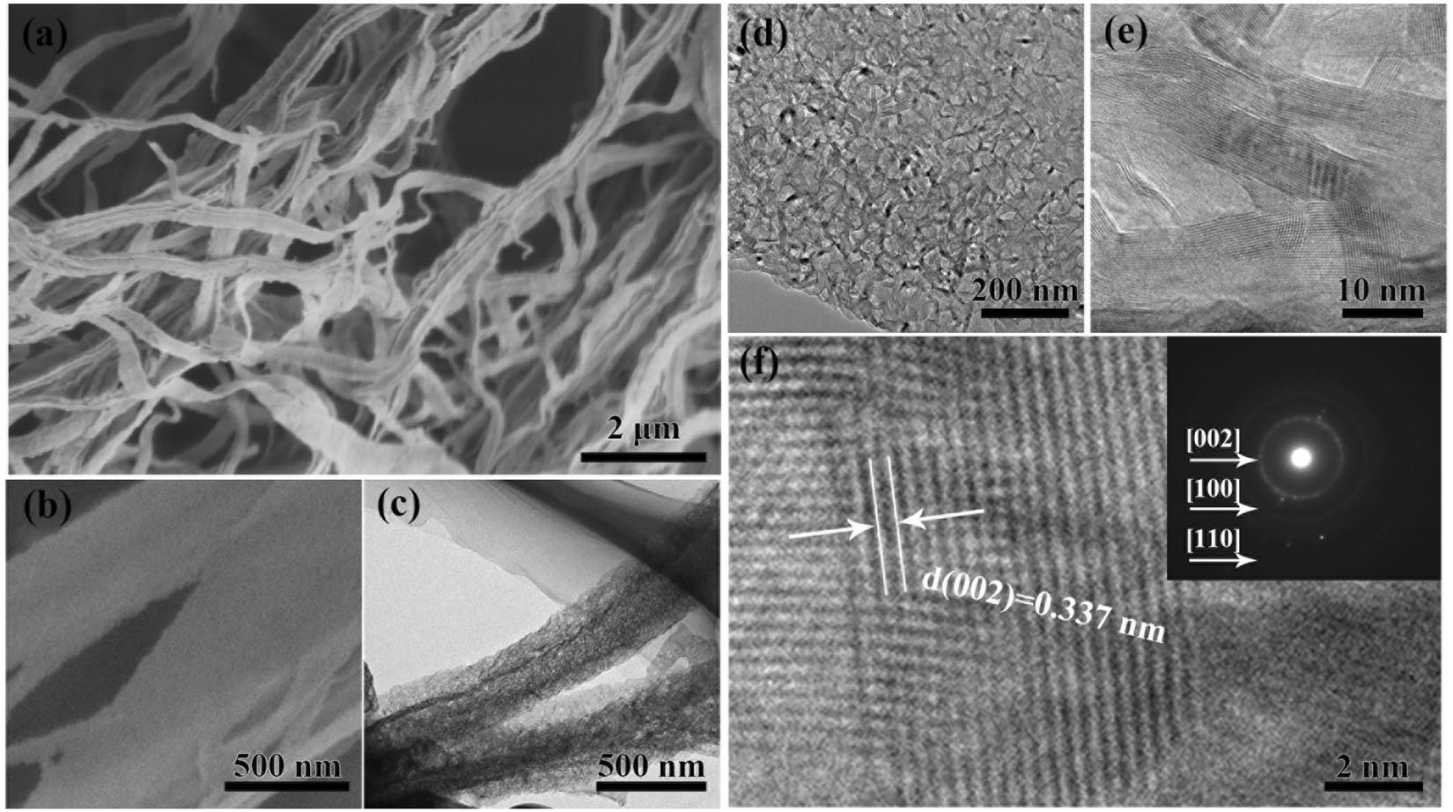
(**a**) SEM image of RLBN. (**b**) High magnification SEM image. (**c**,**d**) Typical TEM images of RLBN. (**e**,**f**) HRTEM image of the RLBN. The layer distance is about 0.337 nm. Inset (**f**) is the corresponding SAED pattern.

Figure 1(a) and (b) are the typical scanning electron microscopy (SEM) images of the synthesized products taken at different magnifications, showing the general pattern, from which a great number of belt-like structures can be clearly observed. The RLBN surface displays a gully morphology, with an ultrathin thickness of 10 nm. The length varies from several to tens of micrometers, and the width is about 300–500 nm. Through TEM images (Fig. 1(c) to (f)), it can be observed that the RLBN is composed of BN nanosheets. Fig. 1(d) shows the arrangement of small nanosheets, which thickness are 8–15 nm, and they present the ultrathin nano banded structure. Figure 1(e) and (f) are two typical HRTEM images of the straight edge, we can observe clearly each wall structure of layered nanosheets, in which a wall thickness of ~7 nm consisting of about 20 layers is clearly seen. The wall is high crystallized with the identical lattice spacing of 0.33 nm (indicators d_(002)_ crystal plane spacing of h-BN).

**Figure 2:**
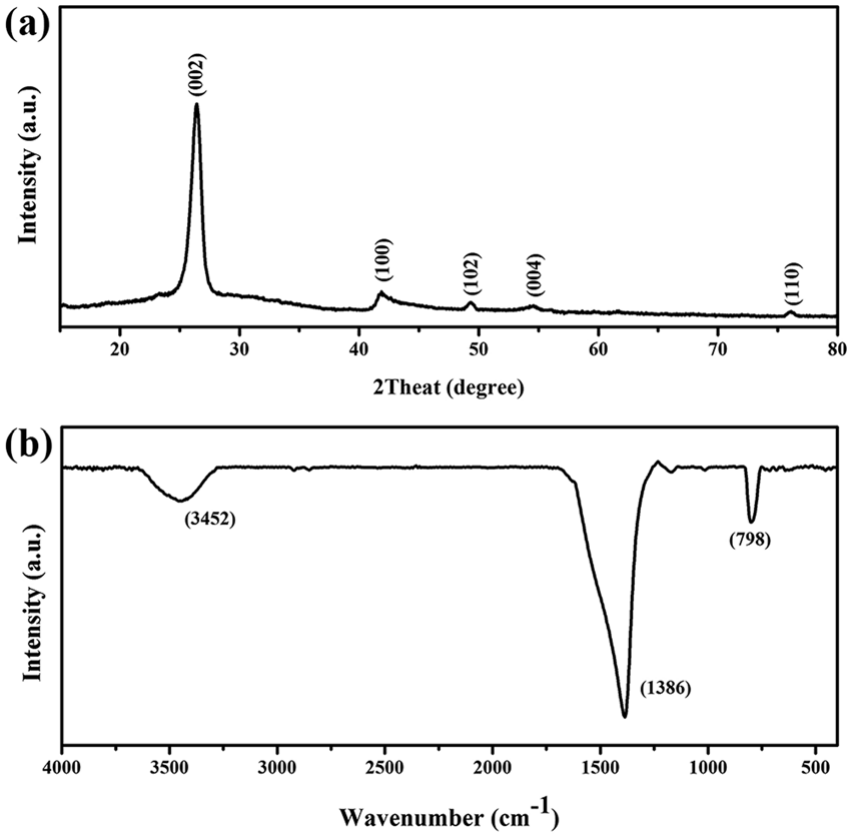
XRD pattern (**a**) and FTIR spectra (**b**) of RLBN.

X-ray diffraction (XRD) result is shown in Fig. 2(a), from where a typical h-BN diffraction pattern with no impurity peak can be observed. Peak located at 2θ = 26.4°, indicating an average lattice spacing of 0.337 nm, is the characteristic (002) diffraction peak. Crystallite size calculated by Full With at Half Maximum (FWHM) of (002) peak using Scherrer Equation, which can be recognized as BN nanosheet’s thickness, is about 10 nm. All these results above are corresponding to ones that obtained from TEM analysis. The FTIR spectra of the samples (Fig. 2(b)) shows two broad peaks around 1386 cm^−1^ and 798 cm^−1^, corresponding to in-plane B-N stretching vibrations mode and out-of-plane B-N-B bending mode of the h-BN materials. The other absorbance peaks are listed as follows, B-OH/B-NH_2_ (3452 cm^−1^), B-N-O (1170 cm^−1^), C-O (1112 cm^−1^), and C-H (2919 cm^−1^ and 2848 cm^−1^).

### Mechanical properties of PHA@BN composites

It is found that the nano structured materials have been considered to the ideal fillers in polymer composites. This is because the nano materials possess significantly high surface energy than the traditional filling materials, the nano materials have dimensions that are much closer to the sizes of atoms, so the cohesive energy can decrease^20, 21^. Nano-BN materials are known to have greatly high modulus and hardness, therefore, they can provide excellent reinforcements to polymers. In this work, 2 wt% of RLBN, P-BN and BNNS were added to the PHA matrix, compared terms of the improvement of mechanical properties.

**Figure 3:**
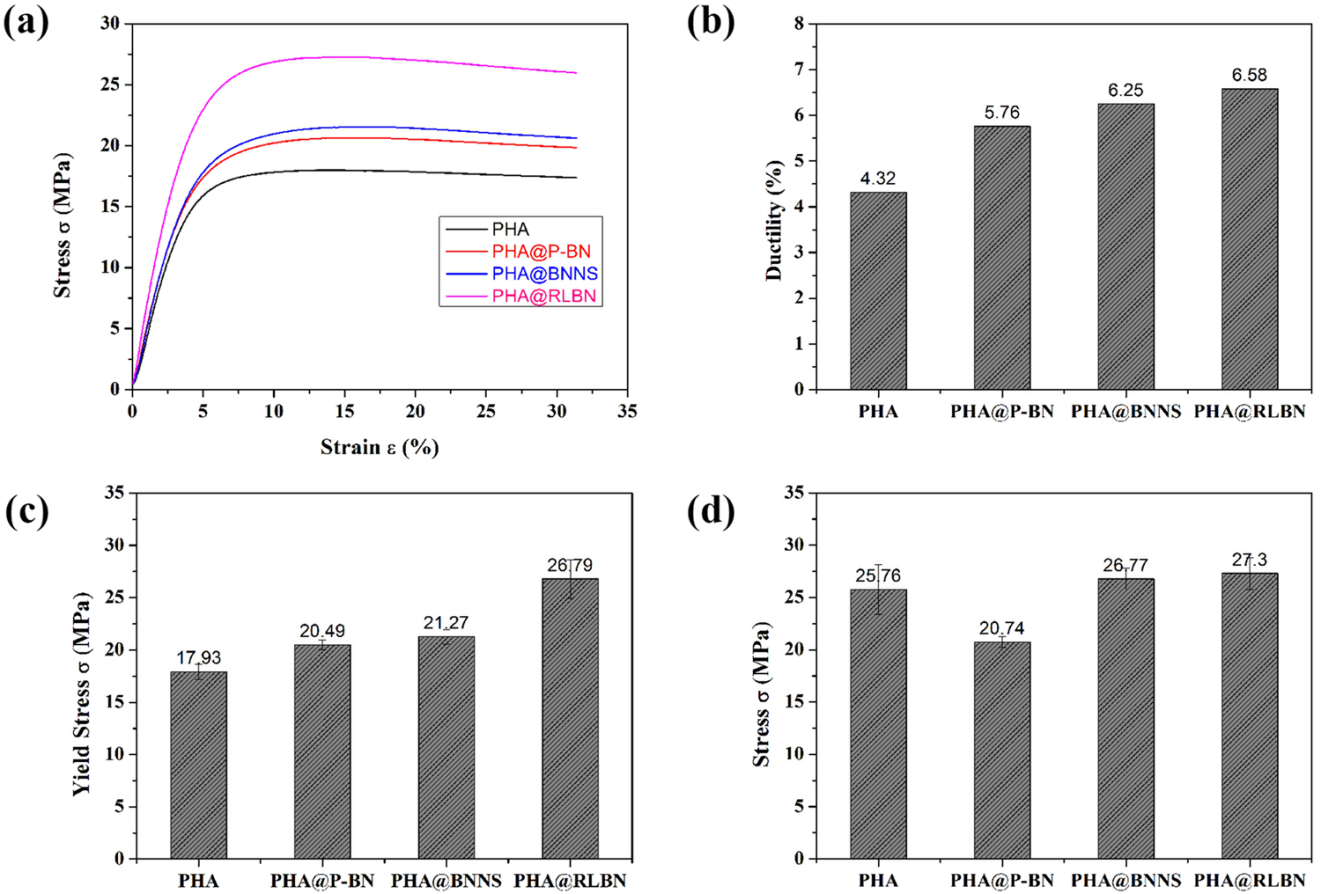
(**a**) Stress-strain curves of PHA and PHA@BN nanocomposites. (**b**)–(**d**) Effect of fillers on mechanical properties of PHA nanocomposites in terms of ductility (**b**), yield stress (**c**) and tensile strength (**d**).

The mechanical properties of the nano-BN composites are examined through standard tensile test. Fig. 3(a)–(d) illustrates the stress-strain curves, strain at break, yield stress and tensile strength of the films along with the standard deviations. Strip-shaped specimens were cut of the solvent cast composite films and five replicates were tested for the PHA resin and each type of composites. Figure 3(a) depicted the typical stress-strain curves of the PHA resin and PHA@BN composites. With the additions of BN at just a minimum amount 2 wt%, the ductility (strain at break) of the composites have been significantly improved, by approximately 33% for PHA@P-BN, 44.7% for PHA@BNNS and 52.3% for PHA@RLBN (Fig. 3(b)). The reason for the improvement in ductility is due to the presence of the shear stress at the filler-matrix interface. The shear stress at the fiber-matrix interface depends on the length of the filler^22^. Thus the 2-D fillers of RLBN are more effective in transferring the stresses and therefor improving more ductility.

The incorporations of nano-BN fillers further increase the composites yield strength and tensile strength (Fig. 3(c) and (d)). The yield strength is seen to increase by 14.2%, 18.6% and 49.4% for PHA@P-BN. PHA@BNNS, and PHA@RLBN, respectively. Besides, the tensile strength shows an increase of 3.9% and 6.01%, for PHA@BNNS and PHA@RLBN, but PHA@P-BN reduces 19.4%, due to the porous BN overall pore too much with low intensity. The RLBN fillers expect correspond to a very high modulus of the composites. A length filler would be more effective in transferring mechanical load from polymer matrix to the filler. The BNNS are known for their extreme hardness and thus are effective in improving the ductility and strength of the composites. However, they are less effective in enhancing mechanical property of the composites as opposed to RLBN. Among the three BN fillers, RLBN seems to be most effective.

### Thermal properties of PHA@BN composites

**Figure 4:**
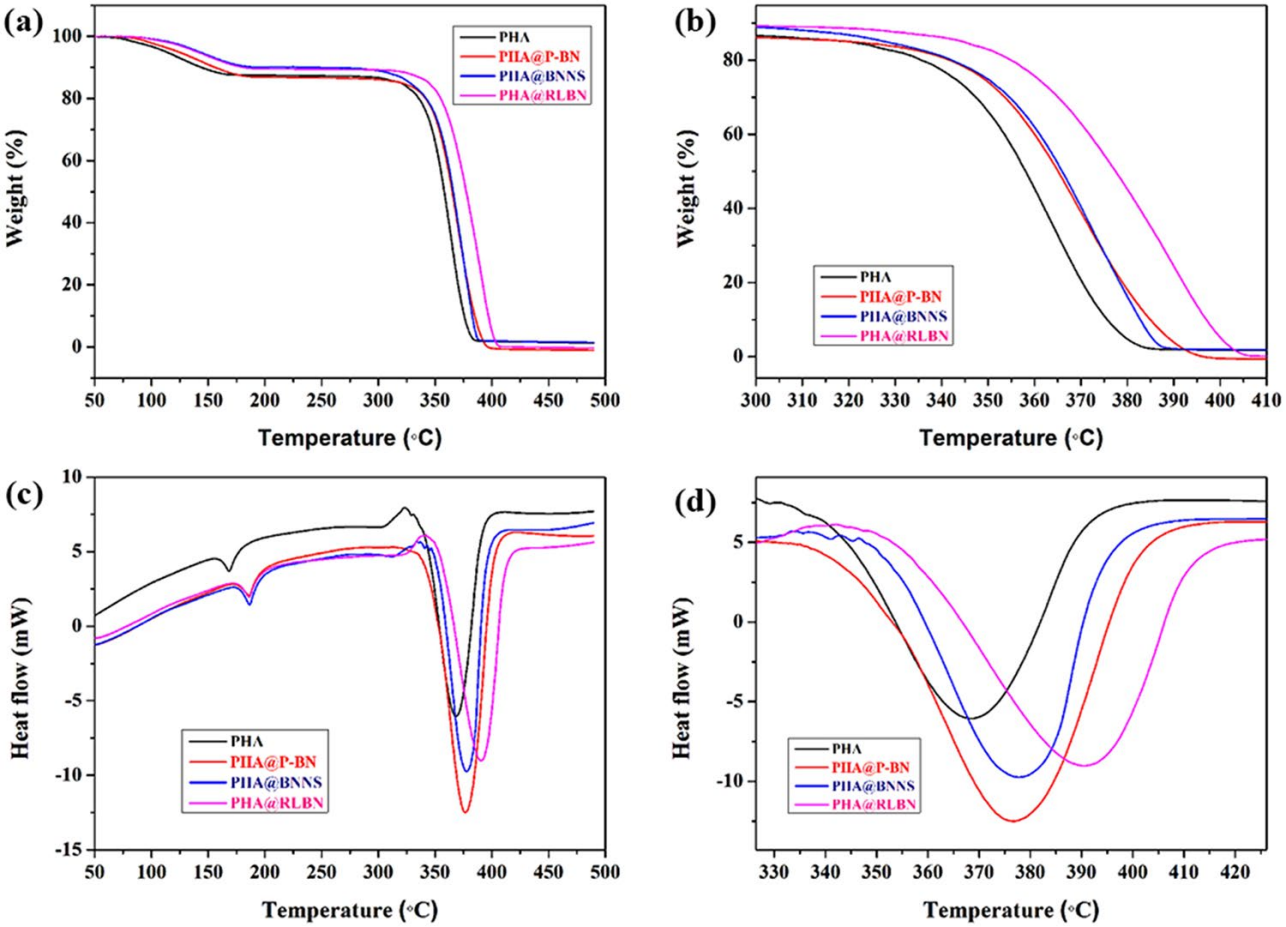
TGA (**a**,**b**) DSC curves (**c**,**d**) of PHA, PHA@P-BN, PHA@BNNS and PHA@RLBN.

Thermal stabilities of the PHA@BN composites were studied by Thermogravimetric Analysis (TGA) and Differential Scanning Calorimetry (DSC). As shown in Fig. 4(a), the composites exhibit two stages of weight losses. Besides, two distinct endothermic peaks at approximately 168 °C and 368 °C, corresponding to each weight loss stage respectively, can be easily observed. The first stage occurs at the temperature below 180 °C. This initial weight loss is rather less, primarily due to the loss of moisture, partial melting of PHA resins and the losses of some surface functional groups. The second stage of weight loss, which can be easily identified between 325 °C~400 °C, corresponding to the decomposition process of PHA resins^23^, is clearly the most significant one. The additions of RLBN in PHA matrices have very increased the decomposition temperatures (T_d_) of PHA resins from 368 °C to 390 °C, showing more effect than that of PHA@P-BN (376 °C) and PHA@BNNS (377 °C).

**Figure 5:**
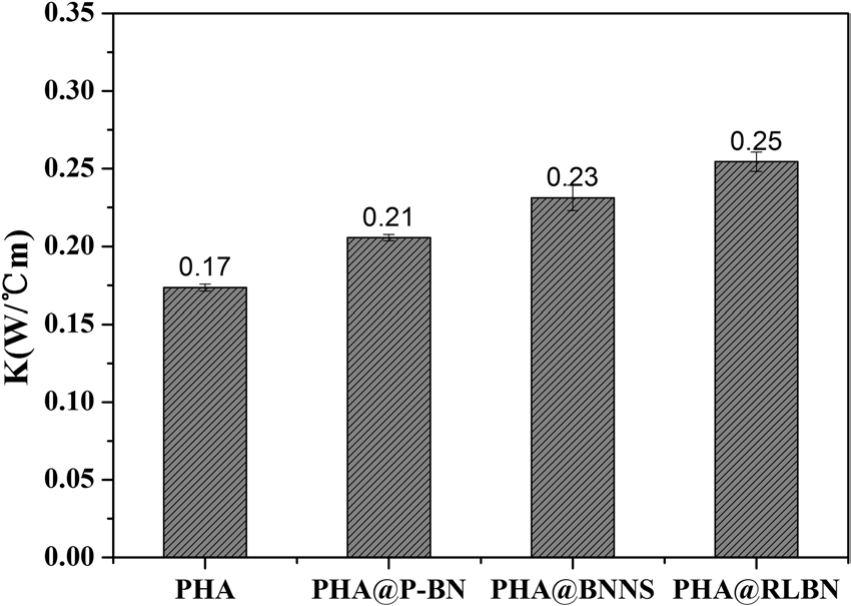
Thermal conductivities of PHA and PHA@BN composites.

Thermal conductivity (Fig. 5) can also be enhanced by addition of RLBN. The thermal conductivity of PHA is about 0.174 W/°Cm, while this value increased by 46.0% when 2 wt% of RLBN added. Furthermore, the thermal conductivity of PHA@RLBN (0.254 W/°Cm) is higher than that of PHA@P-BN (0.206 W/°Cm) and PHA@BNNS (0.231 W/°Cm) at the same loading. It is believed that the large scale 2D RLBN makes the composites easier to form heat conduction channel, which ultimately lead to the enhancement of thermal conductivity in PHA@RLBN composites.

## Conclusions

In conclusion, through a simple self-sacrificed template method, we have successfully synthesized novel RLBN. The RLBN surface displays a gully morphology, with an ultrathin thickness of 10 nm, a length of several to tens of μm, and a width of about 300 to 500 nm. The as-prepared RLBN exhibits excellent performance to enhance the mechanical ability of PHA composite. The ductility, yield strength and tensile strength increased by 52.3%, 49.4% and 6.01% for 2 wt% PHA@RLBN composites, respectively. Furthermore, these RLBN can also be used as the additive in preparing higher thermal stability and thermally conductivity PHA@BN polymer composites. The decomposition temperature (Td) of PHA increases from 368 °C to 390 °C, and the thermal conductivity of PHA@RLBN composites increases by 46.0% than pure PHA resins.

## Methods

### Synthesis of RLBN

In a typical synthesis, a mixture of 0.4946 g boron acid (H_3_BO_3_) and 0.5040 g melamine (C_3_N_6_H_6_) at a molar ratio of 2:1 was dissolved in 800 mL of distilled water, obtain the mixtures of C_3_N_6_H_6_·2H_3_BO_3_ (M·2B). The reaction mixtures (0.005 mol·L^−1^) were heated to 80 °C for 6 h and then cooled to room temperature. The aqueous solution was placed in a refrigerator, frozen into ice with no precipitate generated. The obtained ice block was then dried using a vacuum freeze dryer, owing to the sublimation concentration precipitate to get the white power precursor, i.e. M·2B (C_3_N6H_6_·2H_3_BO_3_) nano-ribbons. The precursor was put in an alumina tube and calcined at 1050 °C for 6 h (in a flow of N_2_ (100 ml/min)) using a resistance-heating horizontal furnace. After cooling to room temperature, the ribbon-like hexagonal boron nitride nano-achitectures were finally obtained.

### Preparation and characterization of RLBN-polymer composites

To generate PHA@RLBN composite film, in a typical synthesis, 2 g PHA was first dissolved in 50 mL chloroform and stirred for 2 h to form a PHA solution. Secondly 0.04 g RLBN were added into PHA solution, and the mixture was stirred for 2 h and then ultrasonic for 2 h at the ice water bath. Thirdly the mixture was agitated for 2 h and at last dried at ambient atmosphere for 48 h. Finally, PHA nanocomposite film containing 2 wt % RLBN was produced. By varying the morphology of BN, a series of PHA@P-BN, PHA@BNNS composites were prepared.

### Characterization

Conventional elemental analyzers (TC500 and CS230, Leco) were applied to analyze the detailed N, O, and C contents, and the remaining percentage is considered as B content. The material structure and morphology of the samples were tested using X-ray powder diffraction (XRD, Purkinje XD2) and field emission scanning electron microscopy (SEM, Dimension 3100 Veeco). FTIR spectra recorded on a Nicolet 7100 spectrophotometer between 400 and 4000 cm-1. Transmission electron microscope (TEM) experiments were performed on a JEOL JEM-2010FEF with an acceleration voltage of 200 kV. Thermogravimetry (TG) and differential scanning calorimetry (DSC) were measured on a DMAX-2500 thermal analyzer from room temperature to 500 °C at a heating rate of 5 °C/min under argon flow. Thermal conductivity was measured by means of a thermal measurement apparatus (LW-9091IR-Series). For the mechanical measurement, the as-prepared films were stretched on a Micro Bionix equipped (MTS Systems Corporation).

## Electronic supplementary material

Supporting Infomation
