# Supplementary material for: Self-sacrificed template synthesis of ribbon-like hexagonal boron nitride nano-architectures and their improvement on mechanical and thermal properties of PHA polymer
Source: Sci Rep. 2017 Aug 21;7:9006. doi: 10.1038/s41598-017-08524-7 (PMC5567015; doi:10.1038/s41598-017-08524-7)
Supplement: Supplementary file 1 — Supporting Infomation [file 41598_2017_8524_MOESM1_ESM.pdf]

Self-sacrificed template synthesis of ribbon-like hexagonal boron nitride nano-achitectures and their improvement on mechanical and thermal properties of PHA polymer

Yan Zhao[1,2], Zhenya Liu[1,2,*], Chaochao Cao[1,2], Chong Wang[1,2], Yi Fang[1,2,*], Yang Huang[1,2], Chao Yu[1,2], Jun Zhang[1,2], Lanlan Li[1,2], Long Hu[1,2] and Chengchun Tang[1,2]

[1]School of Materials Science and Engineering, Hebei University of Technology, Tianjin 300130, P. R. China

[2]Hebei Key Laboratory of Boron Nitride Micro and Nano Materials, Hebei University of Technology, Tianjin 300130, P. R. China

*Phone: +86-22-60202660; fax: +86-22-60202660; E-mail: liuzhenya@hebut.edu.cn (Zhenya Liu); kerrfy@126.com(Yi Fang)

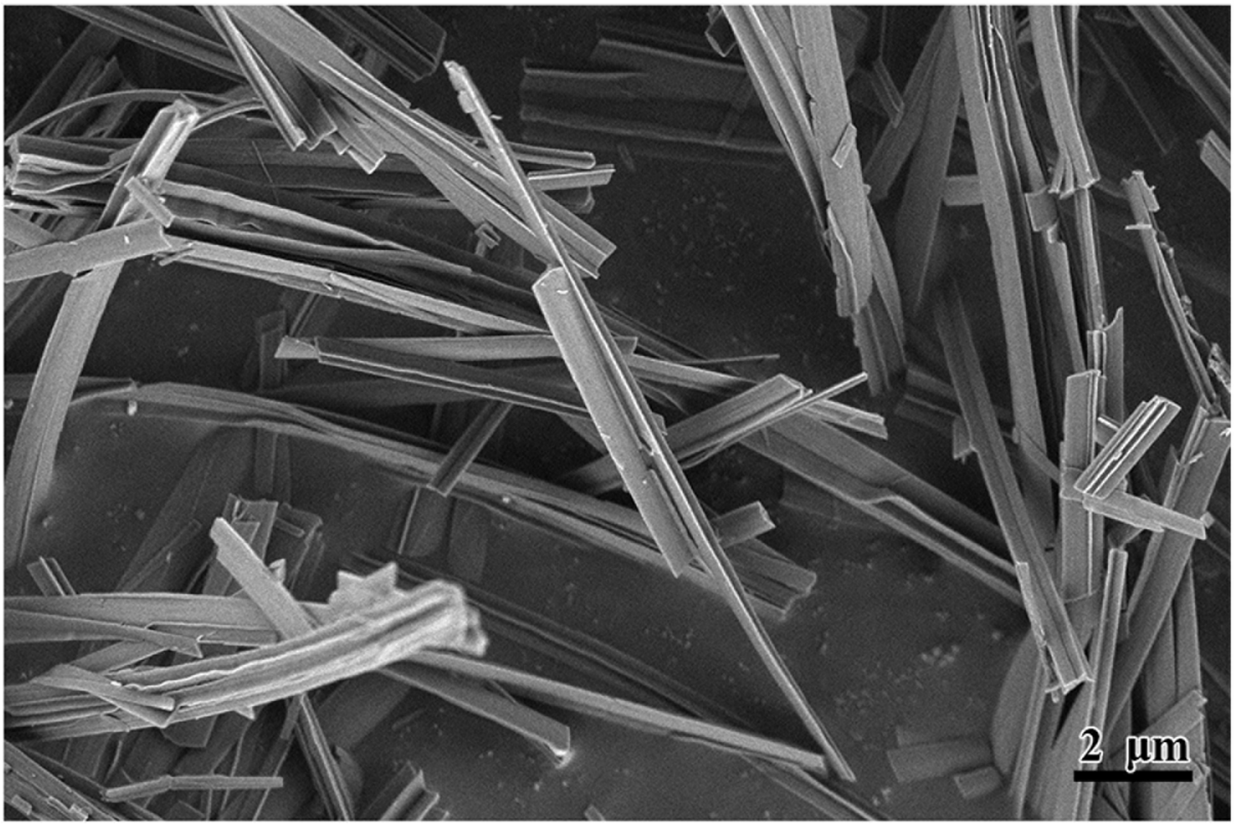

Fig. S1 SEM image of the M·2B ($C_3N_6H_6 \cdot 2H_3BO_3$) precursor, displays 2D ribbon-like morphology with high purity.

The formation conditions of the ribbon-like M·2B nanomaterials are somewhere in between the same process fabricating activated boron nitride[1] and the ultrafine porous boron nitride nanofibers[2]. The ribbon-like M·2B nanomaterials were synthesized by controlling M·2B nucleation and growth kinetics.

1 Li, J. et al. Activated boron nitride as an effective adsorbent for metal ions and organic pollutants. *Sci Rep,* **3**, 3208 (2013).

2 Lin, J. et al. Ultrafine porous boron nitride nanofibers synthesized via a freeze-drying and pyrolysis process and their adsorption properties. *Rsc Adv,* **6**, 1253-1259, (2016).
